# Accuracy of digital templating for total hip arthroplasty: android smartphone and tablet computer versus commercial templating software

**DOI:** 10.1186/s42836-025-00336-9

**Published:** 2025-10-08

**Authors:** Noppadol Wangjiraphan, Charun Sirimongkol, Anuwat Pongkunakorn

**Affiliations:** Department of Orthopaedic Surgery, Lampang Hospital and Medical Education Center, Lampang, 52000 Thailand

**Keywords:** Total hip arthroplasty, Digital templating, PACS, Android, Smartphone, Tablet computer, Accuracy

## Abstract

**Background:**

Preoperative radiographic templating plays an important role in optimizing total hip arthroplasty (THA). Digital templating software ensures precise implant selection, but can be costly and limited to select workstations. A new method using an iPhone/iPad with the picture archiving and communication system (PACS) offers comparable accuracy but is restricted by Apple’s ecosystem. To improve accessibility, we adapted this method for Android smartphones and tablet computers, enabling broader use among surgeons. This study aimed to compare the accuracy and reproducibility of this novel method with a commercial digital templating software.

**Methods:**

Radiographs of 124 hips were retrospectively templated by two independent assessors using three methods. The first used OrthoView® digital templating software. The other two, performed on an Android smartphone and tablet, utilized the PACS measurement tool. A circle was drawn on the acetabular radiograph to represent the cup, then a photograph of the display was imported into Microsoft PowerPoint 365®, where transparent femoral stem templates, scanned from plastic templates, were overlaid. Templating results were compared with implanted cementless THA components for accuracy. Intra-rater and inter-rater reliabilities were analyzed to assess consistency between and within assessors.

**Results:**

Predicting the acetabular cup ± 1 Size could be achieved in 91.1% of cases (113 hips) by OrthoView® and 88.7% (110 hips) by the novel method (*P* = 0.674). The accuracies of three methods were comparable to predict ± 1 size of femoral stem [OrthoView® 90.3% (112 hips), smartphone 85.5% (106 hips), and tablet 87.9% (109hips), *P* = 0.526], and neck length [OrthoView® 94.4% (117 hips, smartphone 91.9% (114 hips), and tablet 93.5% (116 hips), *P* = 0.571]. The neck offset was correctly predicted using OrthoView® in 83.1% (103 hips), comparable with 81.4% (101 hips) using a smartphone and 85.5% (106 hips) using a tablet (*P* = 0.717). No different accuracy was found in each type of the 4 designs of the implanted femoral stems. All methods showed substantial and excellent agreement for intra- and inter-rater reliabilities.

**Conclusions:**

Digital templating for THA using an Android smartphone, tablet, and PACS provides accuracy comparable to commercial software. It is reliable and reproducible for predicting cementless prosthetic size, neck length, and offset across femoral stem types.

## Introduction

Preoperative radiographic templating plays an important role in the success of total hip arthroplasty (THA), aiding in the planned restoration of normal hip biomechanics, optimizing offset, minimizing leg length discrepancies, enhancing joint stability, and reducing the risk of intraoperative fractures [1]. Templating can be conducted through various methods, including two-dimensional (2D) techniques using standard hard-copy radiographs with transparent onlay templates, acetate templates applied to digital screen images, specialized templating software [2, 3], as well as three-dimensional (3D) templating approaches employing advanced digital software based on CT or other imaging modalities [4].

Digital templating involves electronically overlaying implant templates from manufacturers onto digital images in the picture archiving and communication system (PACS), enabling precise selection of implant type, size, and positioning. While its accuracy and reliability for THA are well-documented [4, 5], it may be associated with higher cost and limited availability in some settings. Accessibility may be hindered by software installation restrictions because of a small number of workstations.

A recently developed digital templating method combines the use of an iPhone or iPad (Apple Inc., California, USA) with PACS and has demonstrated accuracy comparable to that of commercial digital templating software [6]. However, this method may not be readily accessible to surgeons who are unfamiliar with or face challenges in accessing the Apple software ecosystem. In response, we adapted this templating method for use with mobile devices (smartphones and tablet computers) running the Android operating system. This study aimed to compare the accuracy and reproducibility of this novel digital templating method with a commercial digital templating system.

## Materials and methods

This retrospective study included patients who underwent primary cementless THA for unilateral hip conditions at our institution between January 2020 and June 2022. All surgeries were performed by two experienced surgeons using the posterolateral approach. Prosthetic components were implanted using a press-fit technique to ensure optimal intraoperative fit.

To evaluate patient eligibility, 1-year postoperative radiographs were analyzed to ensure that the implanted prostheses were appropriately sized and positioned. Anteroposterior (AP) pelvic radiographs were obtained supine, centered on the pubic symphysis, with feet in 15° internal rotation. Proper femoral stem positioning was defined as adequate canal fill or cortical contact at the metaphyseal-diaphyseal junction with < 5° varus/valgus alignment. Optimal neck length and offset were identified by ensuring postoperative leg length discrepancy (LLD) and femoral offset within 5 mm [7]. Proper cup positioning was defined as the center of rotation (COR) within 5 mm of the contralateral femoral head and < 5 mm lateral to Kohler’s line [8, 9]. At the 2-year follow-up, no signs of prosthetic subsidence > 3 mm [10] or loosening were observed in any hip.

**Fig. 1:**
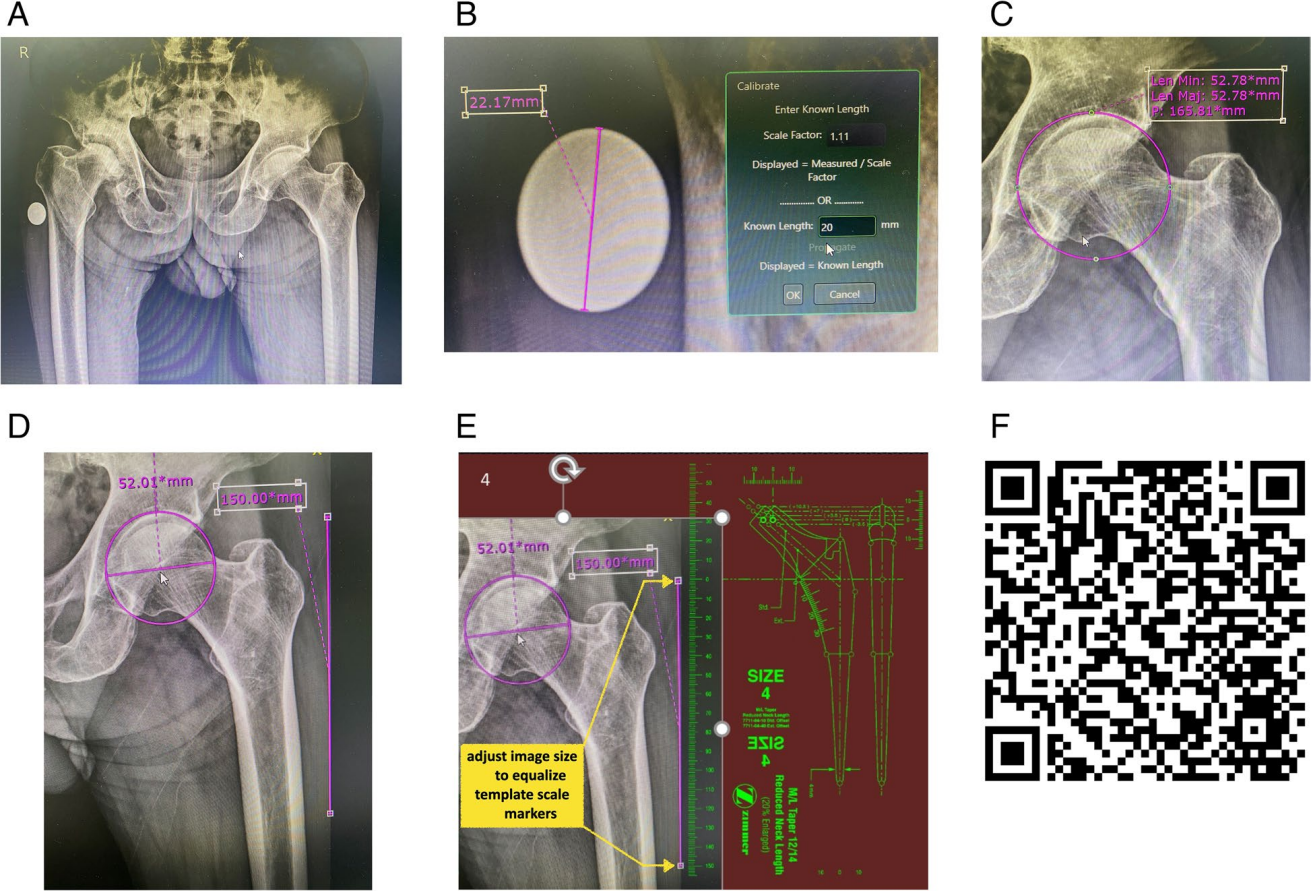
**A** Preoperative standardized pelvic radiograph, showing appropriate rotation of the unaffected femur and a 20-mm calibration coin. **B** Calibration of the coin image to 20 mm using the PACS measurement tool. **C** Placement of a resized circle over the normal acetabulum to simulate cup positioning. **D** Screen image captured using a smartphone or tablet camera, showing the estimated cup Size, center of rotation, and a 15-cm reference line drawn alongside the femur. **E** The hip image is imported into the PowerPoint 365® mobile application and resized so that the 15-cm reference line aligns with the template’s 15-cm scale markers. **F** QR code linking to a Google Drive folder containing video demonstrations of the templating process and scanned digital templates of various femoral stems

Preoperative AP pelvic radiographs were obtained following established guidelines. A 20-mm coin was positioned on the lateral aspect of the greater trochanter for calibration. Radiographs were included only if the thickness of the lesser trochanter on the unaffected hip measured less than 5 mm, ensuring proper femoral rotation [11]. These standardized preoperative radiographs of eligible patients were used to compare the three templating methods evaluated in this study (Fig. 1A).

The first and second methods employed a novel templating technique that shared technical Similarities, differing only in the user interface, which utilized either a smartphone or tablet computer. Both methods consisted of 3 steps.

### Step 1

The femoral prosthesis template was digitized by scanning the manufacturer’s acetate template with a tabletop scanner. The scanned image was processed to create a transparent background and modified using Adobe Photoshop software (Adobe Inc., USA). The outlines and markers were changed to green. Each image of all prosthetic sizes was resized to ensure the scale markers were equalized across all templates. These adjusted templates were then placed on separate slides and centered. The files containing the green transparent templates were downloaded to a smartphone (Huawei P30 Pro 6.47″, China) and a tablet computer (Huawei Mediapad T5 10.1″, China) for viewing and basic editing using the Microsoft PowerPoint 365® mobile application for Android devices (Microsoft Corporation, USA). This cloud-based presentation software offers automatic file saving for convenient access across devices. It provides user-friendly tools for managing images, including inserting photos from the device camera, resizing, and replacing them while preserving formatting. Images can also be aligned, evenly distributed, and layered in front of or behind other elements.

### Step 2

The coin image on the pelvic radiograph was calibrated to 20 mm using the PACS measurement tool (Fig. 1B). A circle was drawn, resized, and positioned on the normal acetabulum according to standard protocol [8] (Fig. 1C). The acetabular cup Size and COR were determined by the diameter and its center. A 15-cm line was drawn beside the femur, and a photograph of the radiograph and line was captured with a smartphone or tablet (Fig. 1D) and imported into the PowerPoint 365® application.

### Step 3

**Fig. 2:**
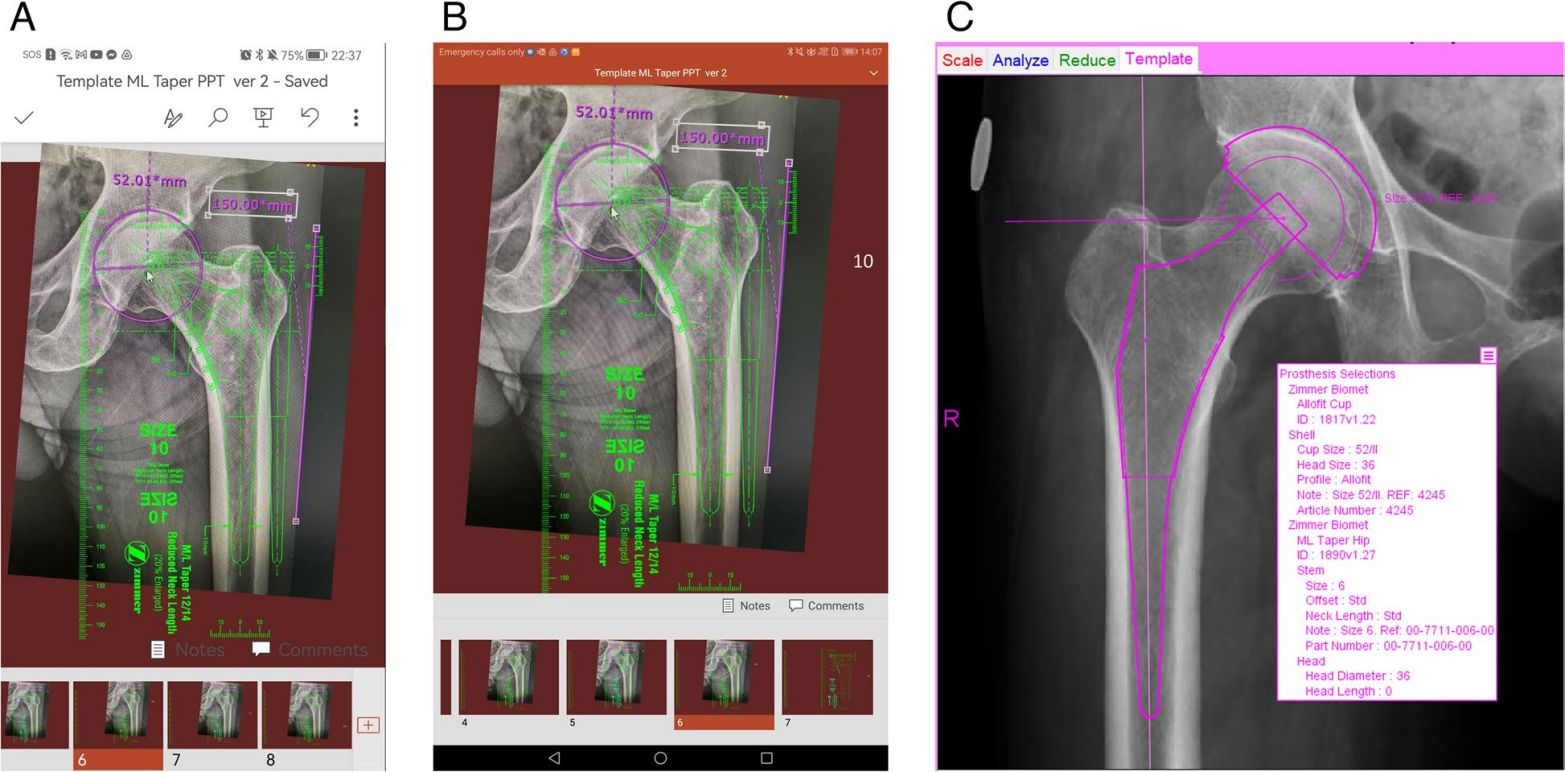
**A** Femoral stem templating using the PowerPoint 365® mobile application on a smartphone. **B** Femoral stem templating using the PowerPoint 365® application on a tablet. **C** Hip radiograph with overlaid digital templates and a summary table of results from OrthoView® software

The photograph was placed behind the transparent femoral prosthesis template. The 15-cm line from the hip photograph was aligned with the template’s 15-cm scale markers by resizing the image (Fig. 1E). The photograph was then positioned using the standard protocol [8]. If the template did not match the femoral canal, the radiograph was copied to the next slides, and the templating process was repeated until the optimal size was selected. A step-by-step instructional video of the templating technique, along with scanned digital templates, can be downloaded from Google Drive by scanning the QR code (Fig. 1F). The final templating result was then recorded using screen capture (Fig. 2A&B).

The third method employed OrthoView® software (Version 7.3.1; Materialise, Belgium) for digital templating, in accordance with standard protocol [12]. Magnification calibration was conducted using a 20-mm coin. An acetabular template was applied to the non-operated hip to identify the COR, followed by alignment of a femoral template to restore hip offset. The resulting images were saved, and templating variables were recorded (Fig. 2C).

Radiographs of each patient were templated using the three methods by two independent assessors—a senior attending staff member and a junior resident in the orthopaedic department, both of whom were not involved in any of the 124 THA procedures and were unaware of the preoperative templating results from the operating surgeons. Prior to the study, both assessors underwent training and standardization by practicing the templating method on 20 patients not included in the study. This training was conducted under the supervision of the most experienced staff member (AP), and the assessors’ results were verified against the actual implanted prosthetic data to ensure quality control.

The templating process was performed twice over a two-week interval. The average of the four templating results was used to predict prosthetic parameters, which were compared with the actual implants. Prosthetic sizes and neck lengths were correct if they matched or were within ± 1 Size, while neck offset types were correct if identical to the offset used in the surgery. To compare templating times, we randomly selected 20 patients, each representing one of four stem types (5 patients per type). Templating durations using the novel method were compared with those using OrthoView®.

Intra-rater and inter-rater reliabilities were assessed using intraclass correlation coefficients (ICCs) for acetabular and femoral prosthesis sizing. Kappa values evaluated the reliability of neck offset and neck length templating. Fisher’s exact test compared prediction accuracies across three methods. Statistical significance was set at *P* < 0.05. Analyses were conducted using SPSS version 11.5 (SPSS Inc., USA).

The sample size calculation targeted detecting a ± 1 Size accuracy difference in femoral prosthesis predictions among templating methods. A systematic review reported 74% accuracy for digital templating software with cementless stems [13]. A 15% accuracy change was deemed clinically significant. Using the two-proportions formula [14] with a 0.05 type I error and 80% power, the required sample Size was 122 hips. The research protocol was approved by the Research Ethics Committee of Lampang Hospital (EC code 60/64).

## Results

During the study, 142 patients underwent unilateral primary cementless THA. Exclusions were 5 hips with intraoperative periprosthetic fractures and 13 hips with postoperative LLD, offset discrepancies > 5 mm, or stem subsidence > 3 mm, resulting in 124 hips from 124 patients. Of these, 66 (53.2%) were women, with a mean age of 56.9 ± 11.0 years (range, 25–79). Diagnoses included femoral head osteonecrosis (49 hips, 39.5%), femoral neck fracture (34 hips, 27.4%), hip dysplasia (14 hips, 11.3%), primary osteoarthritis (9 hips, 7.3%), post-traumatic arthritis (9 hips, 7.3%), rheumatoid arthritis (7 hips, 5.6%), and ankylosing spondylitis (2 hips, 1.6%). Plasmafit cups and Excia stems (Aesculap, Germany) were used in 52 hips (41.9%); Allofit cups and Avenir stems (Zimmer-Biomet, USA) in 48 hips (38.7%); Allofit cups and M/L taper stems (Zimmer-Biomet, USA) in 13 hips (10.5%); and Plasmafit cups and Metha stems (Aesculap, Germany) in 11 hips (8.9%).

**Fig. 3:**
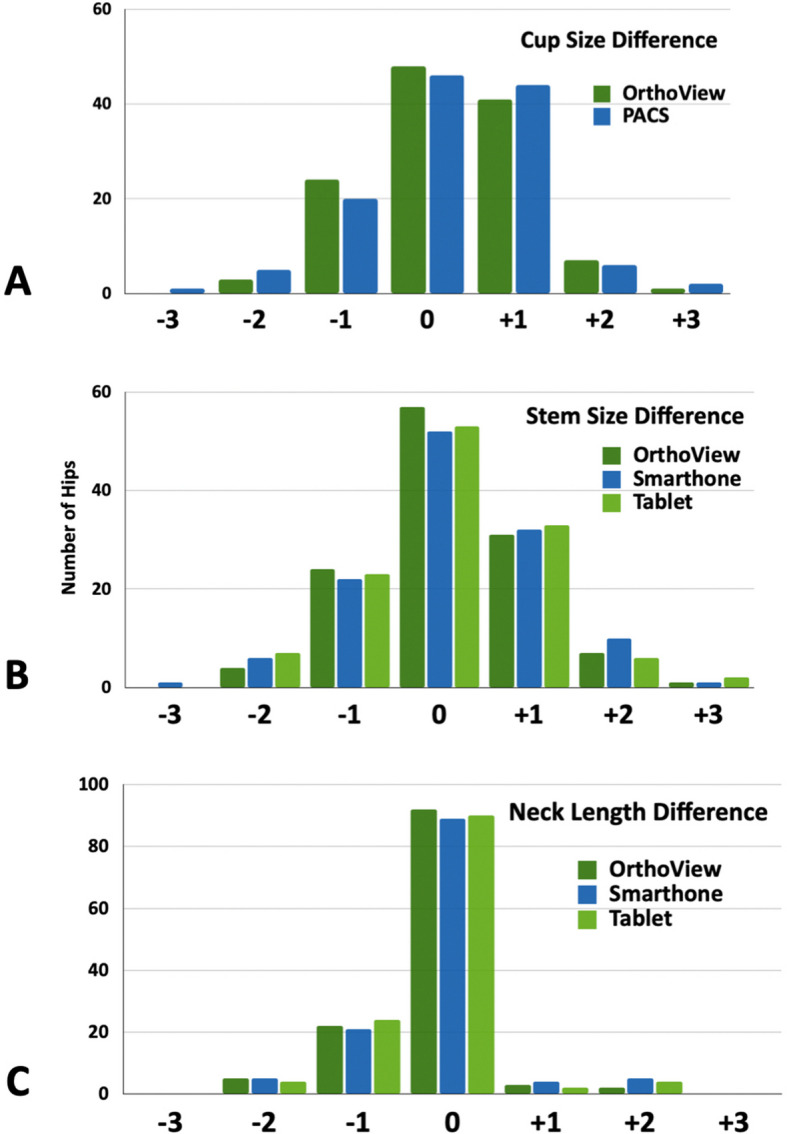
**A** Comparison of acetabular cup size discrepancies between templated and implanted components using the OrthoView® and PACS method. **B** Comparison of femoral component sizes and **C** neck length differences across the three templating methods

The acetabular cup size was predicted within ± 1 Size in 91.1% of cases (113 hips) using OrthoView® and 88.7% (110 hips) with PACS templating (*P* = 0.674). Femoral stem ± 1 Size prediction accuracy was 90.3% (112 hips) for OrthoView®, 85.5% (106 hips) for the smartphone method, and 87.9% (109 hips) for the tablet method (*P* = 0.526). No significant differences were observed across the four femoral prosthesis designs (Table 1). Neck offset predictions were accurate in 83.1% (103 hips) with OrthoView®, 81.4% (101 hips) with the smartphone, and 85.5% (106 hips) with the tablet (*P* = 0.717). Neck length was predicted within ± 1 Size in 94.4% (117 hips) with OrthoView®, 91.9% (114 hips) with the smartphone, and 93.5% (116 hips) with the tablet (*P* = 0.813) (Fig. 3).

**Table 1:** Templating accuracies of the femoral prosthesis of the three methods (*n* = 164)

Femoral components	Accuracy			*P*-value
	**OrthoView** ***n* (%)**	**Smartphone** ***n* (%)**	**Tablet** ***n* (%)**	
Predict size (± 1 size)				
All four stem types	112 (90.3)	106 (85.5%)	109 (87.9%)	0. 526
Excia (*n* = 52)	47 (90.4)	44 (84.6)	49 (94.2)	0.306
Avenir (*n* = 48)	44 (91.7)	43 (89.6)	42 (87.5)	0.941
ML taper (*n* = 13)	12 (92.3)	11 (84.6)	10 (76.9)	0.855
Metha (*n* = 11)	9 (81.8)	8 (72.7)	8 (72.7)	1.000
Predict offset correctly	103 (83.1)	101 (81.4)	106 (85.5)	0.717
Predict neck length (± 1 size)	117 (94.4)	114 (91.9)	116 (93.5)	0.813

The mean templating time was 128 ± 20 s (range, 102–177) using OrthoView®, 238 ± 52 s (range, 155–337) for the smartphone method (including the PACS step), and 200 ± 36 s (range, 99–326) for the tablet method (including the PACS step). Templating for the acetabular cup showed excellent intra-rater and inter-rater reliability for OrthoView® (ICC 0.84 and 0.91, respectively) and PACS methods (ICC 0.89 and 0.86, respectively). Femoral stem size prediction also demonstrated excellent reliability across OrthoView®, smartphone, and tablet methods (ICC 0.84–0.92). Intra-rater reliability for neck offset (Kappa 0.81–0.88) was slightly higher than inter-rater reliability (Kappa 0.71–0.79). All three methods exhibited substantial to excellent agreement for neck length reliability (Kappa 0.74–0.83) (Table 2).

**Table 2:** ICC and Kappa values for intra-observer and inter-observer reliability of templating results for the femoral prosthesis

**Reliability**	**OrthoView**®		**Smartphone**		**Tablet**	
	**values**	**95% CI**	**values**	**95% CI**	**values**	**95% CI**
**Intra-observer**						
Femoral stem size	0.92	0.83–0.98	0.87	0.70–0.92	0.89	0.77–0.97
Offset of femoral stem	0.88	0.75–0.98	0.81	0.66–0.97	0.85	0.74–0.97
Neck length	0.76	0.65–0.86	0.80	0.68–0.94	0.74	0.64–0.85
**Inter-observer**						
Femoral stem size	0.91	0.84–0.96	0.84	0.71–0.95	0.88	0.73–0.96
Offset of femoral stem	0.72	0.63–0.82	0.71	0.56–0.80	0.79	0.71–0.90
Neck length	0.83	0.76–0.91	0.78	0.69–0.87	0.82	0.72–0.93

## Discussion

Currently, digital templating is standard in orthopaedic preoperative planning but often limited by cost and workstation access. A novel method using PACS and Microsoft PowerPoint 365® on handheld devices offers an accessible alternative for restoring personalized hip biomechanics. In this study, templating accuracy was comparable between OrthoView® and the novel method, with results aligning with previous research on implant sizes [6, 13], neck length, and offset [6, 15]. The validity was similar between the smaller smartphone and the larger tablet computer running the Android operating system. The novel method proved effective not only for double-wedge tapered straight stems (Avenir) and tapered, rounded straight stems (Excia) but also for metaphyseal-filling straight stems (M/L taper) and curved, anatomic short stem designs (Metha) in this study.

Plastic templates of acetabular cups can be scanned into digital images using a tabletop scanner and then processed for transparency in Adobe Photoshop [16, 17]. This software allows for line measurements to be calibrated and can serve as templating software, as described by Si et al. (2015) [17]. In contrast, our method utilizes the circle-drawing tool in PACS radiographs to predict cup size, based on the symmetrical hemisphere design commonly used for acetabular cups. Size adjustments are easily made by dragging the mouse to align with the nearest even number for the cup size, without shifting the COR. A diameter line is drawn to form two hemispheres, with the center representing the COR. This circle-drawing technique is similar to the cup templating process in OrthoView®, where the Cup Positioning Wizard automatically places the suggested acetabular template onto the X-ray image. The shared concepts of circles and hemispheres may account for the comparable accuracies observed with PACS and OrthoView® in this study.

For femoral component templating, we used the template manipulation method previously studied in Keynote, a built-in presentation software on mobile devices such as the iPhone and iPad [6]. In this study, the 86–88% accuracy of femoral stem prediction within ± 1 Size using Microsoft PowerPoint 365® was lower than the 91–93% accuracy achieved with Keynote on an iPhone or iPad. This may be attributed to Keynote’s higher efficacy in providing a smooth and precise resizing and rotation function, allowing fine-tuned adjustments when manipulating photographs. This capability enables users to achieve optimal alignment with anatomical structures. In contrast, PowerPoint 365® lacks an image-locking function at the center, and its auto-save feature can cause template misalignments if the template is unintentionally moved or resized, leading to further inaccuracies in subsequent templating procedures. Additionally, the software occasionally becomes unresponsive when copying images into slides, limiting user interaction.

In theory, a larger screen should enhance templating accuracy by providing greater visibility and precision when selecting anatomical landmarks. A larger display allows for more image detail, which could improve measurement accuracy. However, despite the 10.1-inch tablet computer having approximately three times the screen area of the 6.47-inch smartphone, no Significant difference in templating accuracy was observed between the two devices. This finding may be attributed to the extensive magnification capabilities available in the smartphone, which permits up to 540% zoom using a two-finger pinch gesture. Additionally, if further enlargement is needed, a screenshot of the templated radiograph can be taken and viewed in the Huawei Photo Gallery application, providing an extra 7 × magnification. This total magnification, reaching up to 38x, likely compensates for screen size differences, allowing for accurate stem templating.

From the recent systematic review and meta-analysis, the accuracy within 1 Size of digital templating software was 74% (range 53–95%) for cementless stem and 73% (range 41–91%) for cementless cup [13]. Using OrthoView® in this study yielded an accuracy of 83% for offset and 94% for neck length ± 1 Size, comparable to 86% and 89% respectively, in the study of Shemesh et al. (2017) [15]. Whereas its accuracy for prediction ± 1 Size was 90% for femoral stem and 91% for acetabular cup, higher than the range of 60–89% and 70–84% respectively, reported in the previous studies using this software [9, 15, 18, 19]. This is probably from the templating in this study performed in the contralateral healthy Side rather than the operated hip. The normal anatomy provides clear bony landmarks and contour of the acetabulum, facilitating the selection of cup Size to match. Keeping the leg in 15° of internal rotation in a painless hip is much easier than in the painful or arthritic side, leading to a higher probability of obtaining the standardized preoperative radiograph with a suitably rotated femur. Furthermore, we used the latest version of OrthoView®, which allows easier adjustment of the size and characteristics of each component on screen for the optimal fit.

Inclusion in the study was based on 1-year follow-up radiographs, which confirmed that all prosthetic components were appropriately Sized, well-fixed, and free of any Signs of migration or subsidence. Although osseointegration typically completes within 4 to 12 weeks, it can occasionally take up to three years [10]. Nevertheless, subsidence of collarless cementless femoral stems most commonly occurs within the first six months after primary THA [20, 21], supporting the use of a 1-year follow-up as an appropriate time point for assessing early stem stability. To further strengthen the reliability of our findings, all patients were monitored for a minimum of two years to confirm the continued absence of subsidence or prosthetic loosening.

This study has some limitations. First, the stem designs evaluated were tapered straight stems and curved anatomic short stems intended for proximal fixation. As a result, the templating accuracies observed may not be generalizable to other designs, such as diaphyseal scratch-fit stems, cylindrical fully coated prostheses, or modular stems requiring distal reaming. Second, templating accuracy was assessed using a smartphone with one of the largest available screen Sizes in the non-foldable smartphone market, running Android 10 with EMUI 12. The minimum software and hardware requirements for tablets and smartphones include a system partition with at least 2.5 GB of free space and a 64-bit processor. Recommended hardware specifications are a minimum of 2 GB of RAM, 16 GB of internal storage, a 720 × 1,280 pixels (high-definition) display, and preferably a 64-bit CPU. Results may vary when using a smaller screen, lower hardware specifications, a different version of the Android operating system, or a different user interface. Third, we used one senior and one junior assessor, rather than two peer-level assessors, to analyze inter-rater agreement across different levels of expertise. This design aimed to evaluate the usability of the new templating method across varying levels of clinical experience, which enhances the generalizability of the findings. However, using assessors of differing seniority may introduce hierarchical bias and lead to variation in interpretation. To mitigate this risk, all assessments were performed independently, and both assessors were blinded to the preoperative templating records used during surgery. Prior to data collection, both assessors received standardized training, practicing the templating method on 20 patients who were not part of the study, to ensure consistency and quality control. Fourth, we did not directly compare the accuracy of digital templating for THA between the Android smartphone/tablet computer technique and the iPhone/iPad technique because both techniques were not conducted during the same period.

## Conclusion

The digital templating technique for THA using an Android smartphone or tablet computer, combined with a PACS computer system, demonstrated high accuracy comparable to commercially available digital templating software. This technique proved to be reliable and reproducible for predicting cementless prosthetic size, neck length, and offset across different types of femoral stems.

## Data Availability

No datasets were generated or analysed during the current study.

## Abbreviations

THA: Total hip arthroplasty
PACS: picture archiving and communication system
AP: anteroposterior
COR: center of rotation
ICC: intra-class correlation coefficient
